# Spontaneous spike-and-wave discharges during sleep in mice: circadian distribution and impact on sleep quality

**DOI:** 10.3389/fneur.2025.1694773

**Published:** 2026-01-16

**Authors:** Federico Del Gallo, Valentina Salari, Marika Maggia, Manal Salmi, Marina Bentivoglio, Paolo Francesco Fabene, Pierre Szepetowski, Giuseppe Bertini

**Affiliations:** 1 Department of Neuroscience, Biomedicine and Movement Sciences, University of Verona, Verona, Italy; 2 Department of Engineering for Innovation Medicine, Section of Innovation Biomedicine, University of Verona, Verona, Italy; 3 National Institute of Health and Medical Research INSERM, Joint Research Unit UMR 1249, Mediterranean Institute of Neurobiology INMED, Aix-Marseille University, Marseille, France

**Keywords:** sleep-related epilepsy, neurodevelopment, spike-and-wave discharges, animal model, sleep alteration

## Abstract

**Introduction:**

Spike-wave discharges (SWDs) are pathological brain oscillations caused by abnormal thalamocortical synchronization and are a hallmark of several epileptic syndromes. While several experimental models are characterized by SWDs during wakefulness and mimic several key features of absence epilepsy, the spontaneous occurrence of SWDs during sleep has been reported in a limited number of studies. Here, we report a comprehensive characterization of the electrophysiological profile and sleep-wake cycle of a mouse strain previously shown to present sleep-associated SWDs.

**Methods:**

Inbred AJ mice from Jackson Laboratory (JAX) and matched control mice were instrumented for chronic video-EEG/EMG recordings. Data obtained during two 24-hour recording sessions were analyzed to characterize both the sleep-wake cycle and abnormal electrical activity.

**Results:**

Unlike control animals, JAX mice consistently displayed numerous SWDs. The vast majority of episodes occurred during slow-wave sleep (SWS) without overt convulsive manifestations. JAX mice exhibited a reduction in SWS, spent more time in paradoxical sleep, and showed more transitions between vigilance states than controls. Interestingly, SWD events were distributed in a circadian fashion, peaking around the end of the rest period.

**Discussion:**

Alongside previously characterized models, the consistent and spontaneous occurrence of SWDs during SWS makes the JAX mouse a viable experimental model to understand the mechanisms behind sleep-related SWDs. The results, including the peculiar circadian distribution of SWDs, pave the way for further studies addressing a fundamental pathogenetic conundrum, i.e., why is epileptiform activity specifically concentrated in SWS.

## Introduction

Spike–wave discharges (SWDs) are abnormal patterns of brain electrical activity associated with several epileptic conditions. The electroencephalographic (EEG) signature of SWDs is a sustained cycle of sharp spikes, each followed by a wave, indicating a brief period of cortical inhibition, with a typical frequency of 3–4 Hz in humans (1). SWDs recorded bilaterally in the EEG are the hallmark of childhood absence epilepsy (CAE) and are a central feature in other neurological conditions including Lennox–Gastaut syndrome, Landau–Kleffner syndrome (LKS), and continuous spike and wave discharges during sleep (CSWS), where the discharges are specifically concentrated during slow-wave sleep (2, 3).

Several experimental models of spontaneous SWD syndromes have long been available. Among them, two well-established genetic models - the Rijswijk Wistar-Albino-Glaxo rat (WAG/Rij) (4) and the Genetic Absence Epilepsy Rats from Strasbourg (GAERS) (5)–exhibit spontaneous bilateral SWDs within the 7–11 Hz frequency range, closely mirroring those observed in CAE. In addition, models such as the “tottering” and “stargazer” mouse strains exhibit SWDs typically in the 5–7 Hz range (6, 7).

These models have been instrumental in outlining hypotheses regarding the circuits and mechanisms that lead to the abrupt transition from normal brain waves to this specific type of paroxysmal activity (5, 8). A leading hypothesis suggests that SWDs result from abnormal synchronization within thalamocortical circuits, facilitated by enhanced corticothalamic feedback and altered inhibitory tone (9). While the relative contribution of the cortex and the thalamus as initiators of discharges has long been a matter of debate, an emerging consensus points to SWDs as a common pathological outcome of various cellular and molecular anomalies affecting different components of the circuitry (10).

This view is supported by the recent development of realistic neural-network models of thalamocortical circuits, which are able to generate patterns of oscillatory activity that faithfully reproduce normal phase transitions (11). Interestingly, SWD-like signals could be reproduced in the network by altering parameters corresponding to known distinct molecular anomalies and pharmacological manipulations (12). For instance, increasing tonic GABA_A_ inhibition in thalamocortical neurons led to 4 Hz spike-and-wave discharges resembling absence seizures, highlighting the impact of disrupted inhibitory balance in subcortical regions. Similarly, a selective reduction in cortical phasic GABA_A_ inhibition was sufficient to induce pathological oscillations, reflecting how certain pharmacological interventions can destabilize network dynamics and promote seizure-like activity (11). It is evident that simulation studies must be grounded in, and validated by, robust experimental evidence. While numerous lines of research have explored the mechanisms underlying SWD generation in absence epilepsy, comparatively little attention has been directed toward the neurological conditions in which SWD complexes are specifically linked to sleep. Elucidating this association is particularly important within the framework of the emerging hypothesis that phenotypically similar SWDs may arise from distinct combinations of brain states and underlying anomalies.

Electroencephalographic patterns resembling CSWS have been observed following unilateral single freeze lesions in the somatosensory cortex of wild-type neonatal mice (13), and we have previously reported the spontaneous occurrence of numerous SDWs in juvenile *Grin2a* knock-out (KO) mice, a genetic model of sleep-related epilepsy (14, 15).

Another strain of mice, the inbred AJ mouse from Jackson Laboratory (AJ JAX),^1^ has been shown to spontaneously exhibit SWDs, mostly during slow-wave sleep (SWS, the murine homolog of NREM sleep), while no abnormal brain electrical activity is observed in the same mouse strain acquired from a different vendor (formerly Harlan Laboratories and Envigo, currently Inotiv’s AJ OlaHsd)^2^ (16). The only published report, however, did not provide an in-depth electrophysiological characterization and left several important questions unanswered, including the stability of the inbred phenotype over time, the relationship between abnormal electrical activity and the physiological alternations between vigilance states, or the quality of sleep.

An in-depth description of the clinical profile of these rodent models is a necessary step toward the wider goal of unraveling the mechanisms of SWD generation, which could be accomplished through a combination of experimental and network simulation approaches. In addition, such characterizations could lead to the adoption of these mouse strains as experimental models of sleep-related epilepsy belonging to the family of epileptic encephalopathies.

Here we present the results of a study comparing AJ JAX mice, affected by SWD activity, to control animals. Long-term electroencephalographic (EEG) and electromyographic (EMG) recordings were used to fully characterize the animals’ electrophysiological and sleep phenotype over the course of two full days. During the same period, the sleep–wake cycle was scored in order to correlate the occurrence of SWDs with specific vigilance states and to detect possible alterations in the architecture and overall quality of sleep in affected animals.

## Materials and methods

### Ethics statement

All experiments were approved by the local Animal Care and Use Committee (Interdepartmental Centre for Experimental Research, CIRSAL, University of Verona) and authorized by the Italian Ministry of Health (protocol n°239/2016-PR), in strict adherence to the European Communities Council (86/609/EEC) directives and the ARRIVE guidelines, minimizing the number of animals used as well as their suffering.

### Animals

Two cohorts of 4-week-old male inbred AJ mice were purchased, respectively, from Jackson Laboratories (here referred to as JAX) and Inotiv (ex Harlan Laboratories and Envigo, AJ OlaHSD, here referred to as OLA). Another cohort of age-matched, male C57BL/6 J mice (here referred to as C57), was acquired from Charles River. For the purpose of the current study we selected male subjects in order to reduce oestrus-related variability of results. In any case, the previously reported electrical abnormalities were present in both male and female JAX mice (16). Upon arrival, animals were housed for at least 3 weeks under veterinarian control in the animal facilities at standard temperature and humidity (23 ± 1 °C and 60 ± 5% respectively) and an inverted 12 h/12 h light/dark cycle. *Zeitgeber* time (ZT) 0, corresponding to lights-on time, was set at 6:00 p.m. Food and water were available *ad libitum*.

### Surgery

Groups of adult (3–8 month-old) mice of all strains (JAX, *n* = 16; OLA, *n* = 8; C57, *n* = 16) were implanted with chronic recording electrodes. Briefly, a pair of EMG electrodes (stainless steel wires) were inserted into the left nuchal muscle and stitched in place. EEG electrodes were represented by 4 stainless-steel screws threaded into holes drilled through the skull in the right frontal, right parietal, left parietal, and occipital bones, at commonly adopted stereotaxic coordinates (17–21). Mice were allowed to recover from surgery for at least 14 days. See Supplementary Information, section M1 for further details.

The relatively long delay between electrode implants and the initial recording sessions allowed for full clinical recovery. All animals appeared in good health and did not display overt behavioral anomalies.

### *In vivo* video-EEG/EMG recording

Recording chambers were 30 × 30 × 55(h) cm plexiglass cages (PhenoTyper®, Noldus Information Technology, Wageningen, The Netherlands) equipped with food and water dispensers. An infrared-sensitive, monochrome video camera attached to the top lid allowed continuous on-line monitoring of the animals’ conditions and spontaneous behavior, minimizing the stress due to the presence of researchers in experimental rooms. EEG/EMG signals were amplified, filtered (EEG: high-pass at 0.3 Hz; low-pass at 70 Hz; EMG: high-pass at 10 Hz; 50-Hz notch filtering for both signals), and digitized at 400 Hz. See Supplementary Information M2 section for more details.

Video-EEG/EMG recordings lasted 7 days without interruption, starting at the end of the post-surgical recovery period, with mice left undisturbed in the recording chamber. EEG and EMG signals were devoid of signs of irritative activity related to the surgical intervention. The analyses reported here refer to the first (D1) and the seventh (D7) day of EEG/EMG recording.

### Sleep–wake analysis

Vigilance states were determined by visual scoring of the EEG (ipsilateral frontal–parietal derivation) and EMG traces in 10-s epochs. Arousal states were defined as wakefulness (WAKE), slow-wave sleep (SWS), and rapid eye movement (REM) sleep based on changes in multiple parameters, including EEG and EMG appearance and EEG power density estimation, as described previously (20) (see Supplementary Information M3 section for details). Any movement artifacts or electrical noises in the recordings were tagged and the corresponding epochs excluded from subsequent analyses.

### Detection and analysis of epileptiform events

**Figure 1:**
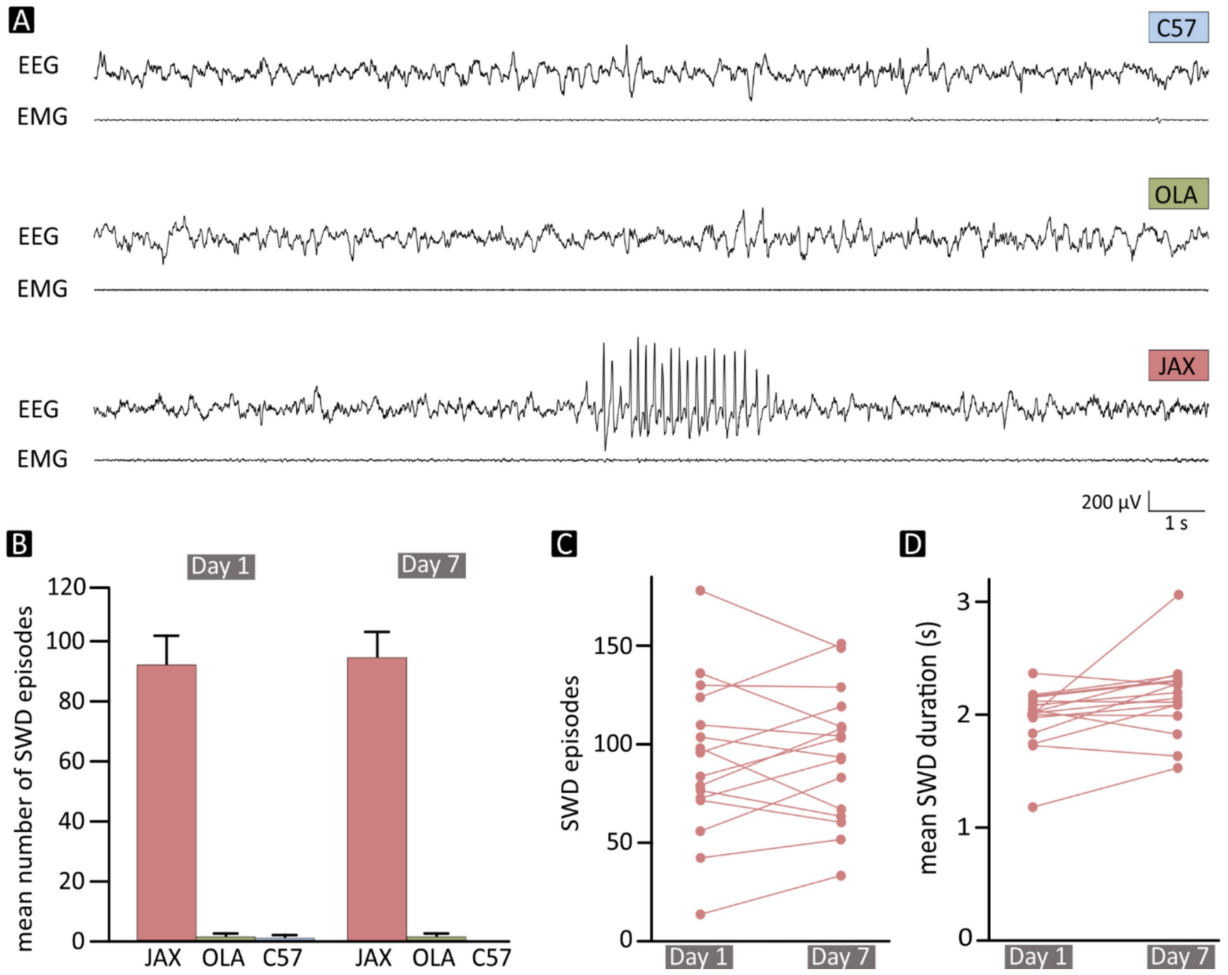
**(A)** Normal slow-wave sleep EEG/EMG sample epochs in two control mice (C57 and OLA) and a single, 4-s-long spike–wave discharge (SWD) contained in a sample trace from a JAX mouse. **(B)** Mean number of SWD episodes /24 h. at recording days 1 and 7, for each of the three mouse groups. **(C)** Total number and **(D)** mean duration of SWD events for each recorded JAX mouse. EEG, Electroencephalogram; EMG, Electromyogram; C57, C57BL/6 J mice; JAX, AJ mice from Jackson Laboratory; OLA, AJ mice from Inotiv (ex Envigo).

**Figure 2:**
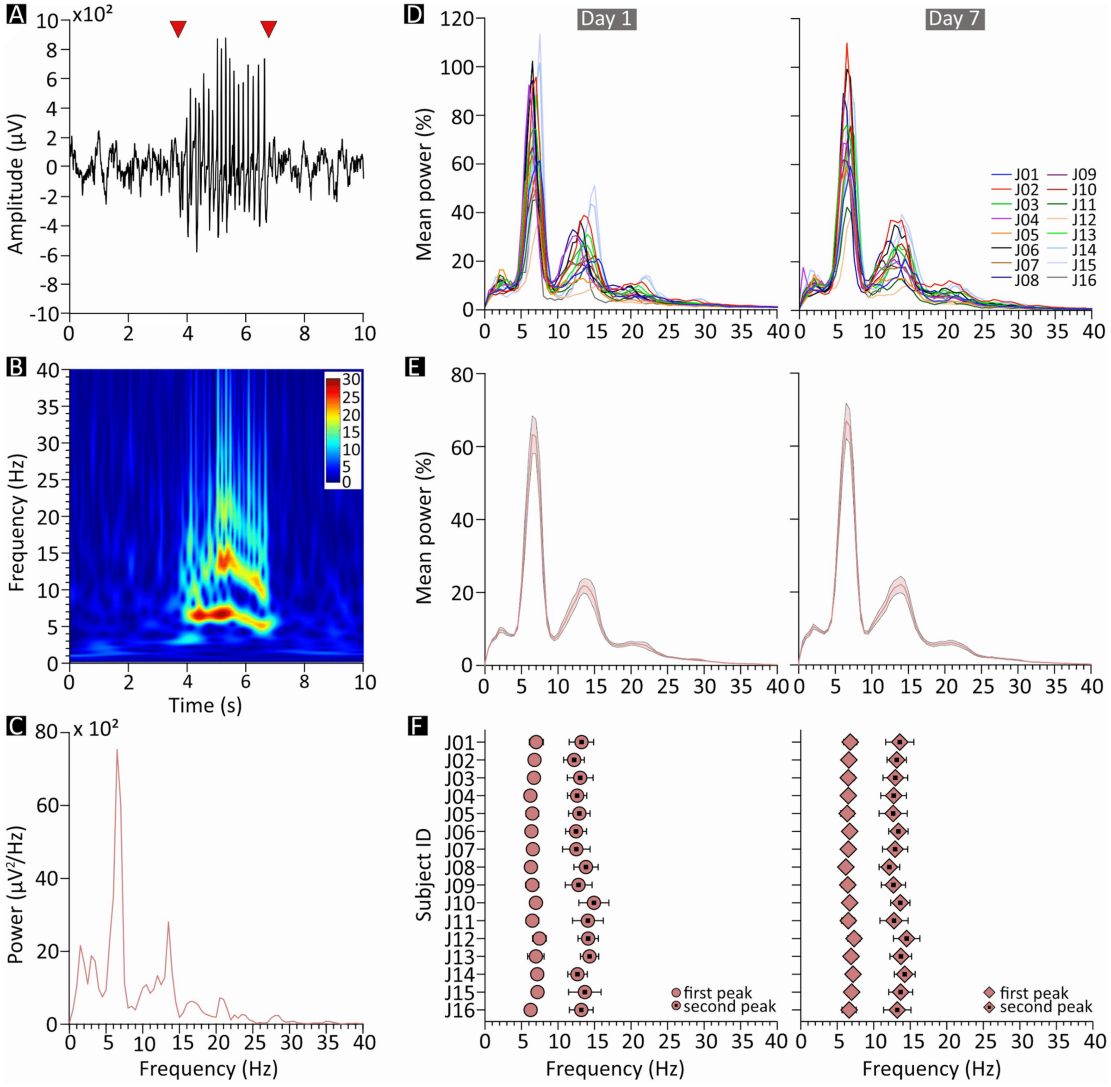
Spectral features of spike–wave discharges (SWDs) in JAX mice. **(A)** Example of a 10-s epoch of slow-wave sleep containing a single SWD episode (starting and ending at the arrowheads). **(B)** Spectrogram of the same sample epoch; pseudocolors represent power in arbitrary units. **(C)** Power spectrum of the same epoch. **(D–F)** Charts on the left- and right-hand side describe results from recording days 1 and 7, respectively. **(D)** Mean of all normalized SWD power spectra, plotted separately for each JAX mouse and expressed as percentage of the total power. **(E)** Population average of the plots in **D**. **(F)** Mean frequency, for each JAX mouse, of the two most prominent power peaks revealed by the plots in **D**. JAX, AJ mice from Jackson Laboratory; Hz, Hertz; ID, identity number; 𝜇V, microvolts.

We define spike–wave discharges (SWDs, Figure 1A) as a high-voltage (at least 1.5-fold increase relative to background EEG), sharp discharge pattern of at least 4 cycles (14, 16). SWDs were detected in the EEG frontal derivation with an overly permissive automated detection algorithm written in MATLAB® (Mathworks, Natick MA, United States) followed by visual inspection to remove false-positive events. The beginning and end of each SWD episode were marked (Figure 2A), so that EEG segments limited to the duration of the discharge could be extracted. From each segment, the power spectral density (PSD) was estimated using Welch’s overlapped-segment averaging estimator, with a 2-s moving Hanning window and 0.5-s overlap and normalized against the PSD obtained from the total, artifact-free EEG (14, 16).

### Statistical analysis

Data are reported as mean ± SEM values. An alpha level of *p* < 0.05 was used to detect statistically significant differences.

Unless otherwise stated, all comparisons between mouse strains (JAX, OLA, C57) and between recorded days (D1, D7) were analyzed with mixed-model ANOVAs, which reported the main effects of “strain” (the between-subject factor) and “day” (the within-subject factor), as well as the “strain*day” interaction. Post-hoc tests for the “strain” factor were conducted with Tukey’s HSD test, while pairwise comparisons between days were Bonferroni-corrected.

To analyze the distribution of SWD events during each day of recording, the Rayleigh test and relative circular statistics were used to evaluate the uniformity of event distribution (MATLAB’s *circHist* function (22, 23)), followed by cosinor analysis (the least squares fit of a 24-h cosine curve) in order to assess the circadian distribution of SWD events (24) [MATLAB’s *cosinor* function (25)].

The results for each of the performed statistics are described in detail in Supplementary Information, sections R1-R16.

## Results

The relatively long delay (14 days) between electrode implants and the initial recording sessions allowed for full clinical recovery and resulted in EEG and EMG signals devoid of signs of irritative activity related to the surgical intervention. Aside from the SWD episodes described below, cortical activity in JAX mice was indistinguishable from that of controls.

### All JAX mice display SWDs

Numerous SWDs were detected in all JAX mice (Figures 1A, 2A), while OLA and C57 animals were nearly unaffected, with only occasional discharges in some of the subjects. With no significant differences between D1 and D7 (on average, ~93 over the 24 h, Figure 1B). In contrast, OLA and C57 control animals were nearly unaffected. On average, OLA animals had ~1.5 discharges per 24 h (Figure 1B), while C57 mice were virtually SWD-free. For statistical details, see Supplementary Information, section R1 (SI–R1).

Interestingly, while the number of SWDs in JAX mice showed substantial variability between subjects, only moderate within-subject changes were observed from D1 to D7, indicating a fairly stable picture for each animal (Figure 1C). The mean duration of SWD episodes (Figure 1D) was relatively stable both within (D1-D7) and between JAX mice. Aside from SWDs, cortical activity of JAX mice was indistinguishable from that of controls. Due to the exiguous number of episodes in control animals, all subsequent analyses on electrical abnormalities were limited to the JAX strain.

In the JAX group, the between-subject variability in the number of detected SWDs *per* day was evaluated as standard deviation from the mean: 39.95 at D1 and 33.82 at D7. The coefficient of variability, i.e., the standard deviation/mean ratio, was 43.51% at D1 and 35.62% at D7; only moderate within-subject changes were observed from D1 to D7 (on average, standard deviation/mean ratio: 18.39%), indicating a relatively stable picture for each animal.

### SWDs consistently peak in the theta range

For each JAX mouse, we computed the frequency spectrum of the EEG signal encompassing each individual SWDs (e.g., the fragment between the red arrowheads in Figure 2A). The sample spectra in Figures 2B,C clearly shows a major peak in the theta range, representing the fundamental frequency of the discharge, as well as a second peak at approximately twice the frequency. Moreover, SWD episodes were occasionally preceded by a brief period of oscillations in a similar frequency range.

Both peaks were reliably found in all subjects, at an average frequency of around 6.7 and 13.3 Hz, respectively (Figure 2F). The first peak was especially predictable (D1: 6.73 ± 0.10 Hz, D7: 6.69 ± 0.07 Hz, Figures 2E,F; Supplementary Figure S1), while the second peak was only slightly more variable (D1: 13.28 ± 0.19 Hz, D7: 13.27 ± 0.15 Hz, Figures 2E,F; Supplementary Figure S2). Peak frequencies were also remarkably consistent within subjects (Figure 2D) and did not change noticeably between D1 and D7; indeed, a qualitative inspection suggests that the variability observed in peak frequency is mostly due to between-subject differences, while within-subject changes from D1 to D7 (Figure 2D) were especially modest.

### SWD events mostly occur during SWS

**Figure 3:**
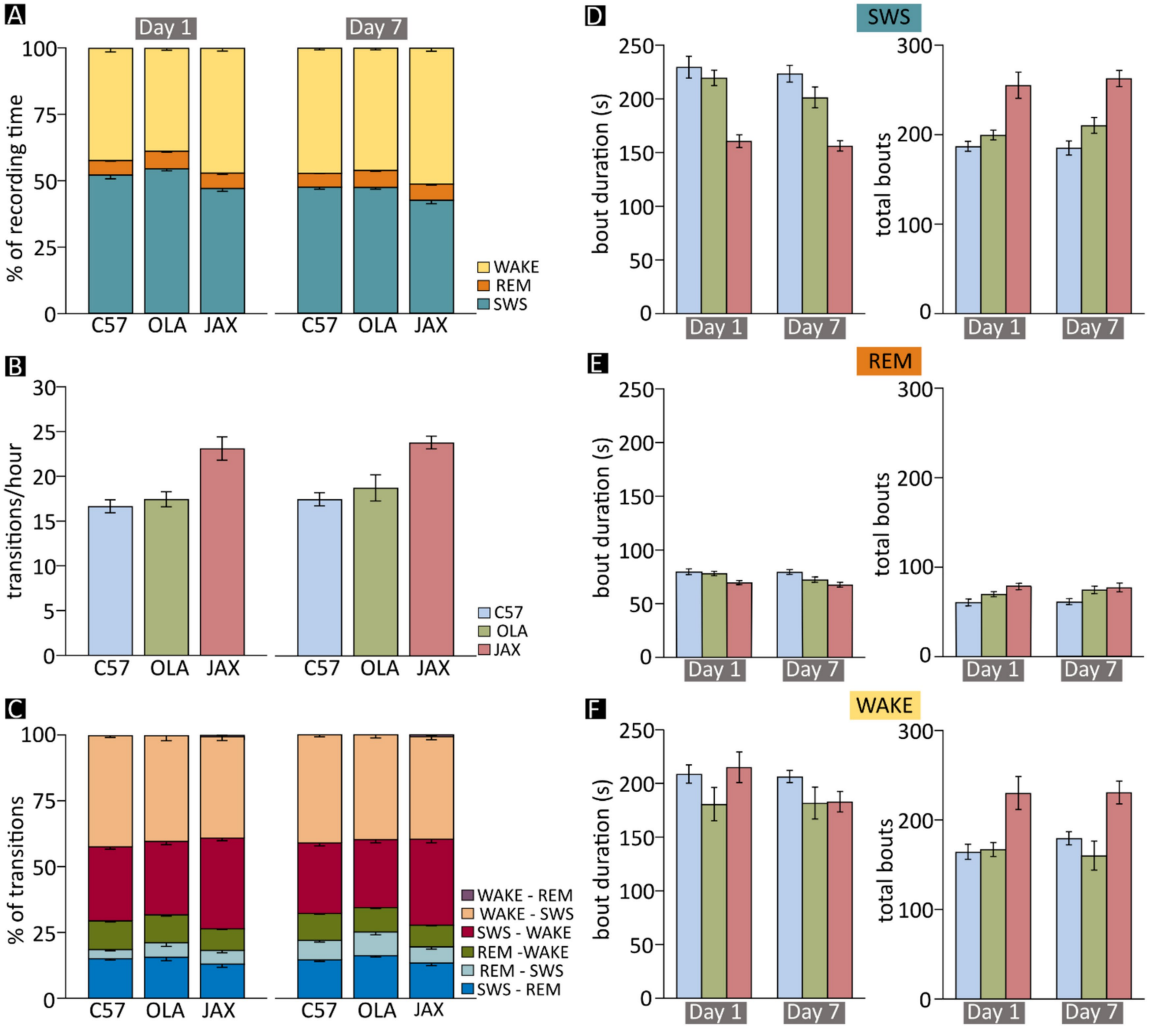
Sleep–wake analysis in C57, OLA and JAX mice. Left- and right-hand side charts show results for recording days 1 and 7, respectively. **(A)** Mean fraction of time spent in each vigilance state during the 24 h of recording. **(B)** Mean frequency of transitions between vigilance states (all combinations). **(C)** Mean proportion of transitions between each dyadic combination of vigilance states. **(D–F)** Mean duration and total number of bouts of slow-wave sleep (SWS) **(D)**, REM sleep (REM) **(E)**, and wakefulness (WAKE) **(F)**. C57: C57BL/6 J mice; JAX, AJ mice from Jackson Laboratory; OLA, AJ mice from Inotiv (ex Envigo).

**Figure 4:**
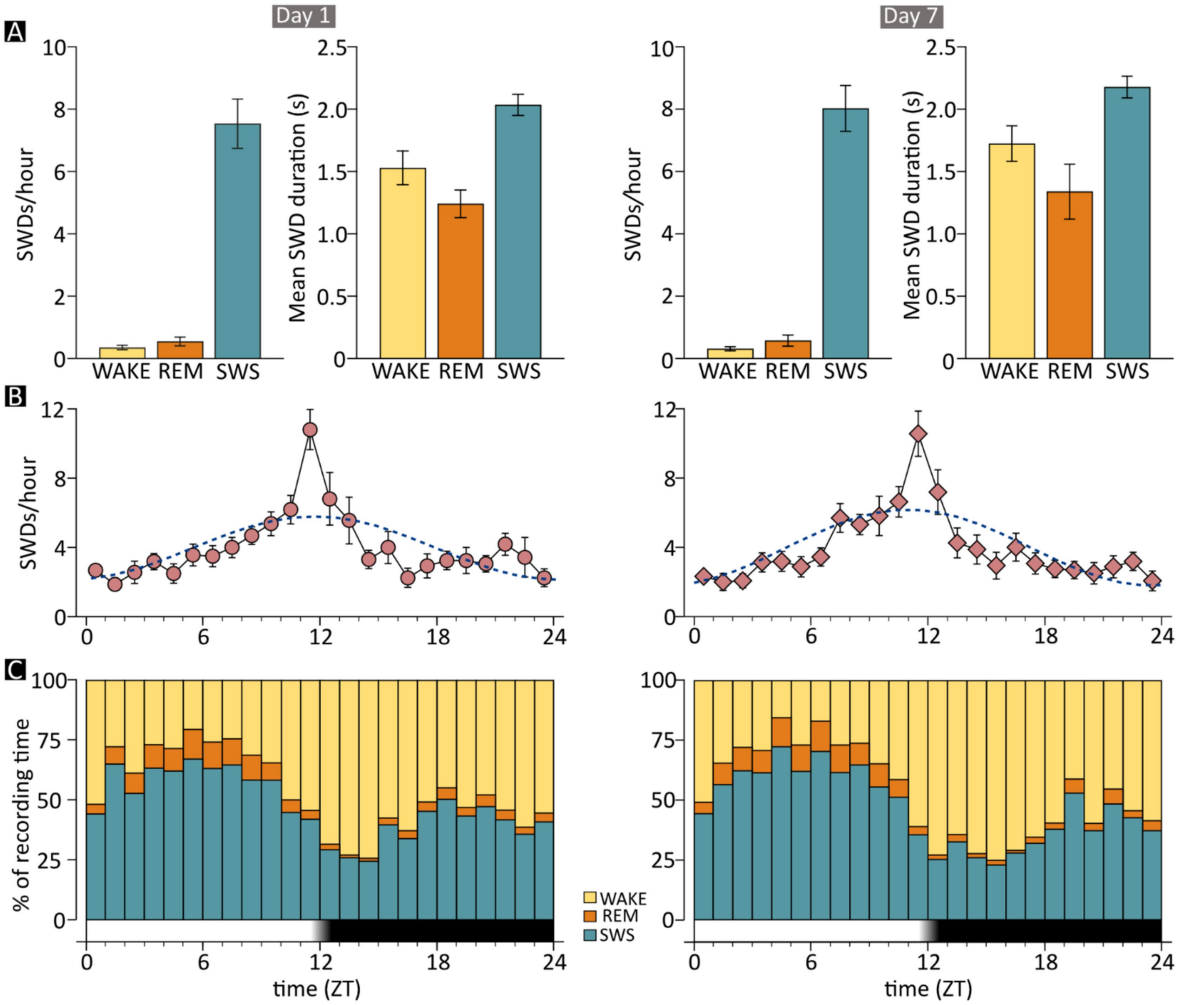
Occurrence of spike–wave discharges (SWDs) by time of day and vigilance state in JAX mice. Charts on the left- and right-hand side show results for recording days 1 and 7, respectively. **(A)** The mean frequency and duration of SWD episodes are shown separately for the three examined vigilance states. Frequencies are computed as the number of episodes *per* hour spent in each state. **(B)** The mean frequency of epileptic events is shown on an hourly basis, together with the cosinor function (dotted line). **(C)** The mean fraction of time spent in each vigilance state, for each hour of the recording day. The time axis in B and C shows the number of hours elapsed from “lights-on” time. The dark band indicates the “lights-off” hours. REM, REM sleep; SWS, slow-wave sleep; WAKE, wakefulness; ZT, Zeitgeber time; JAX, AJ mice from Jackson Laboratory.

On average, mice spent roughly 50% of their time in SWS and approximately 5% in REM (Figure 3A). The three vigilance states, however, were distributed according to the normal circadian rhythm (Figure 4C), with animals spending more time asleep in lights-on conditions (ZT 0–12), compared to the behaviorally active, lights-off period (ZT 12–24).

As seen in human patients with ESESs, SWD events in JAX mice occurred almost exclusively while animals were in SWS (D1: 93.57% ± 1.56%, D7: 94.62% ± 1.08% of total SWD events, Figure 4A). Only about 5% and less than 1% of all discharges occurred during WAKE and REM sleep phases, respectively. See Supplementary Information, section R2 for details.

Furthermore, the above-mentioned small increase in SWD duration between D1 and D7 was independent of state. See Supplementary Information, section R3 for details.

### SWD events show a circadian distribution

The distribution of SWD episodes varied systematically throughout the day. The mean number of events grew steadily during the light period, reaching its maximum near the transition between the light and dark phase (ZT11 - ZT13), then declined progressively until it reached the same low point observed at the beginning of the day (Figure 4B). The circadian pattern was statistically robust (see Supplementary Information, section R4) and was nearly identical between recording days (Figure 4B, left vs. right).

### SWS is reduced in JAX mice

The high prevalence of SWDs in SWS prompted the possibility of a correlation between epileptiform activity and the sleep–wake cycle. Therefore, we assessed both qualitatively and quantitatively the pattern of vigilance-state alternations in each of the strains at D1 and D7. On average, the time spent in SWS was significantly shorter for JAX compared to OLA or C57 mice (12 and 10% less, respectively, Figure 3A and Supplementary Information, section R5). In addition, all three strains displayed a moderate decrease of total SWS time from D1 to D7 (Figure 3A and Supplementary Information, section R5).

Especially considering the “polyphasic” nature of mice, who present numerous state transitions during the 24-h period (26, 27), the above-described shifts in the total amount of vigilance states do not entirely capture the nature of the alteration. Thus, we further examined the architecture of each vigilance state by evaluating the number of occurrences (hereafter referred to as “bouts” and defined as periods of at least 10 s spent in the same vigilance state) and their duration.

The reduction of time spent in SWS by JAX mice was mainly due to the decrease in the mean duration of SWS episodes during both D1 and D7 (Figure 3D and Supplementary Information, section R6). This reduction was only partially compensated by an increase in the number of bouts in JAX vs. control mice (Figure 3D and Supplementary Information, section R7).

### The architecture of REM sleep shows between-strain variations

While the amount of time spent in REM sleep did not change significantly between experimental groups or recording days (Figure 3A and Supplementary Information, section R8), the average duration of REM sleep bouts was significantly shorter in JAX mice compared to C57 mice (Figure 3E and Supplementary Information, section R9), while the results for OLA mice fell in between. Conversely, REM sleep bouts were significantly more numerous in JAX than in C57 mice (Figure 3E and Supplementary Information, section R10). Again, OLA animals showed intermediate average values (Figure 3E).

### JAX mice spend more time in wakefulness

After excluding the occasional artifactual epoch, the time spent in wakefulness vs. sleep is obviously mutually dependent. Thus, as expected, JAX mice spent significantly more time awake, both at D1 and D7, compared to control mice (10% more than C57 mice and ~15% more than OLA mice, Figure 3A and Supplementary Information, section R11). This was mainly due to a higher number of WAKE bouts (see Supplementary Information, section R13), while the mean duration of such bouts was comparable between strains and between recording days (Figure 3F and Supplementary Information, section R12). Additionally, all three strains displayed a moderate increase of the total time spent awake from D1 to D7 (Figure 3A and Supplementary Information, section R11), as a complementary effect of the already mentioned decrease in total SWS time (Figure 3A).

### Sleep quality is disrupted in JAX mice

A high degree of sleep fragmentation, expressed as the frequency of vigilance state transitions over time, is a commonly adopted indicator of poor sleep quality (17, 20, 28). Indeed, sleep in JAX mice was significantly more fragmented compared to control strains (Figure 3B and see Supplementary Information, section R14). We also hypothesized that the effect could be specifically ascribed to transitions into and out of SWS, given the high prevalence of SWD episodes. Thus, we computed for each animal the relative frequency of each possible transition pair (Figure 3C) and found that JAX mice had significantly more SWS → WAKE (awakening) and fewer REM sleep → WAKE transitions compared to controls (Figure 3C and Supplementary Information, section R15).

**Figure 5:**
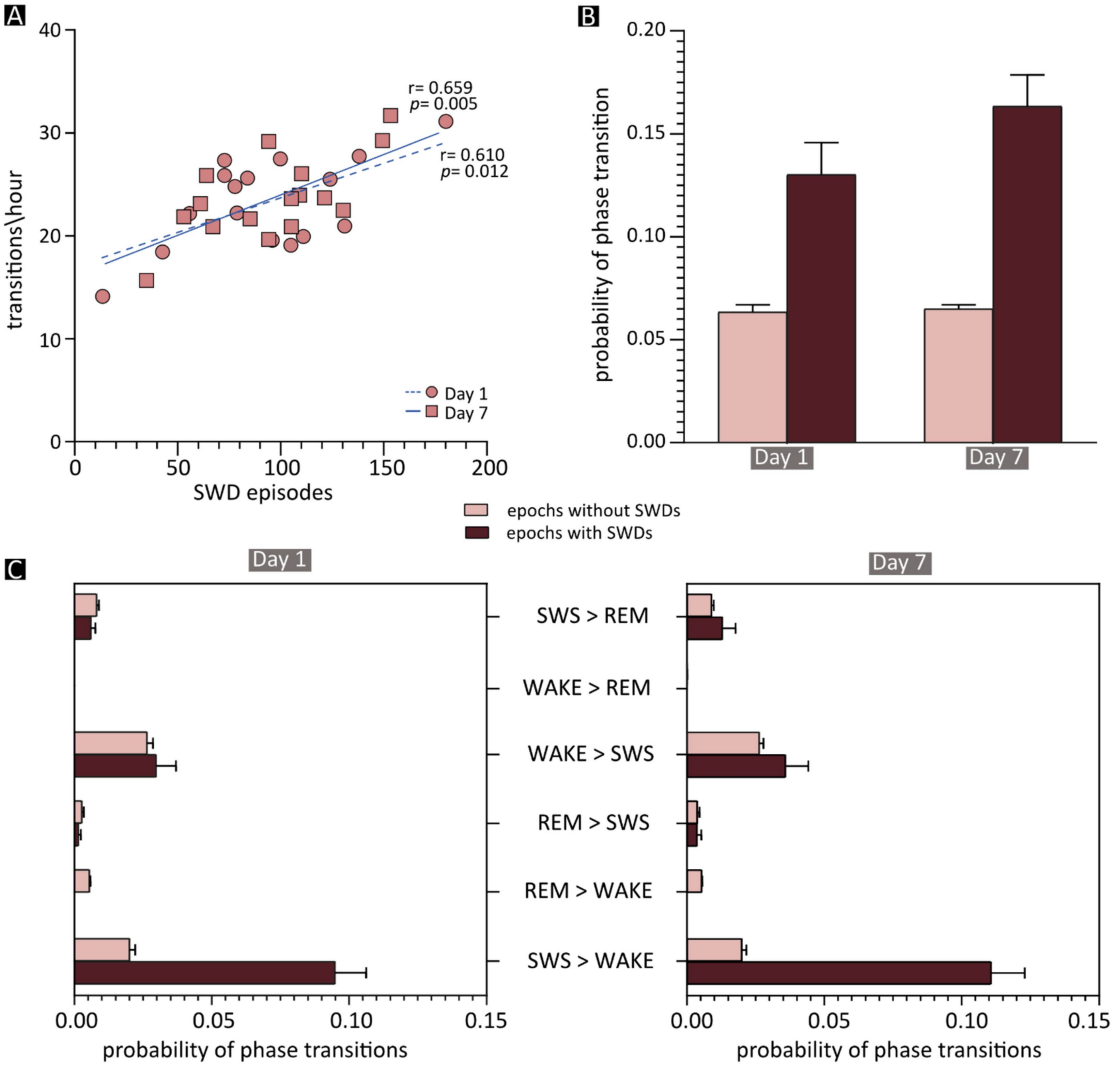
Effects of spike–wave discharges (SWDs) on phase transitions. **(A)** Correlation between the frequency of vigilance-state transitions and the total number of SWD episodes in each JAX mouse, shown separately for recording days 1 and 7. The results of Pearson’s correlation statistics (see also Supplementary Information, section R16) are shown next to the two regression lines. **(B)** Mean probability of a phase transition occurring after an epoch containing an SWD episode (dark red) or after a “normal” epoch (light red). **(C)** The mean probability of each phase transition after an SWD-containing (dark red) or -free (light red) epoch during the first (on the left) and the seventh (on the right) day of recording. REM, REM sleep; SWS, slow-wave sleep; WAKE, wakefulness; SWDs, spike–wave discharges.

We then tested whether the amount of SWS disruption in JAX mice depends on the severity of the epileptic disorder and found that the number of transitions and the number of SWDs are indeed positively correlated (Figure 5A and Supplementary Information, section R16).

This correlation opens the possibility that individual SWD episodes may be directly responsible for state changes. To evaluate this, we computed in JAX mice the frequency of state transitions occurring after the 10-s epochs containing at least one SWD episode, compared to epochs devoid of epileptiform activity. On average, state transitions were more than twice as likely to occur following SWD-containing compared to SWD-free epochs (Figure 5B and Supplementary Information, section R16). As might be expected, the vast majority of such transitions consisted of awakenings from slow-wave sleep (SWS → WAKE, Figure 5C).

## Discussion

Here we report a highly consistent and specific pattern of spontaneous spike-and-wave discharges in all JAX mice studied so far, while abnormal events were extremely rare in control animals. The sleep–wake cycle was also altered in JAX mice, with reduced slow-wave sleep and poor sleep quality compared to the control groups.

### Spike–wave discharges in JAX mice

The pathological discharges (~2 s-long on average, with a main frequency peak around 7 Hz) closely resembled those originally reported for this strain (16), demonstrating that in the intervening years the inbred phenotype has not been diluted. The morphology, spectral components, and duration of the SWD episodes observed in the present study share significant similarities with those described in several other rodent models of epilepsy.

For example, several known spontaneous mutations in mice are associated with SWDs resembling those observed in JAX mice. Among these, at least 4 (the so-called “tottering,” “lethargic,” “ducky,” and “stargazer”) share both genotypic and phenotypic features (7, 29, 30), including ataxic gait, paroxysmal dyskinesia, and absence-like seizures with SWDs between 5 and 7 Hz (6). Also, mutations in the human GABA_A_ receptor’s 𝛼1 subunit (GABRA1) are associated with absence epilepsy, and mutant mice lacking the *Gabra1* gene display brief SWDs often accompanied by behavioral arrest (31). Although not reported quantitatively, discharges in this KO model appear to be in a similar frequency range as those observed in JAX mice. Similar SWDs have also been observed in the WAG/Rij and GAERS rat strains (32, 33), which are well-established rodent models of spontaneous absence epilepsy. Interestingly, SWDs in human patients have a typical frequency around 3 Hz. To our knowledge, however, in all the above-mentioned rodents the spectral peak of the discharges is between 6 and 9 Hz, possibly due to fundamental inter-species differences (34).

The occurrence of brief SWDs with the same morphological characteristics were also observed in rodent models of other neurological conditions, including female mice carrying the genetic defect known to cause Rett syndrome, who display spontaneous epileptiform discharges with a main frequency of 5–8 Hz (35–37), and hypocretin-deficient narcoleptic mice, characterized by short, high-amplitude bursts of pointed theta waves (7 Hz) occurring during REM sleep or accompanying cataplexy, similarly to what happens in narcoleptic patients (38). Finally, high-amplitude polyphasic complexes lasting 0.3 to 4.6 s with a main spectral frequency around 7–8 Hz were also found in mice carrying the homolog human mutation associated with the familial form of Creutzfeldt-Jakob disease (CJD) (39).

### Timing of SWDs in the sleep and wake cycle

While the electrical properties of SWDs are substantially shared among multiple rodent models, the timing of the discharges in JAX mice stands out from the others. In particular, the vast majority of episodes in JAX mice (94% of the total) occurred during NREM sleep, i.e., on top of a synchronized EEG pattern, were rare in wakefulness (5%), and virtually absent during REM sleep (1%). In contrast, SWDs observed in several other models are (1) concentrated during wakefulness (32, 40, 41), or during REM sleep (38), (2) more or less equally present during the different vigilance states (39, 42), or (3) their distribution was not quantified (31).

To our knowledge, two other mouse models show this close temporal association between SWS and SWDs. First, in a model of focal cortical dysplasia (FCD), a single freeze lesion in the right somatosensory cortex of neonate wild-type mice induced, later in life (5 months and older), non-convulsive SWDs in the 4–12 Hz frequency range, occurring predominantly during sleep episodes (13). More recently, we investigated *in vivo* the electrical phenotype of mice lacking the GluN2A subunit of NMDA receptors. Pathogenic variants in the corresponding *GRIN2A* gene represent the most frequent known cause of the epilepsy-aphasia spectrum of childhood epilepsies, and epileptic encephalopathies with speech and language dysfunction (43) includes ESES. We reported that *Grin2a* KO mice studied at 1 and 2 months of age spontaneously presented numerous SWDs peaking at 12–13 Hz (14). Interestingly, this abnormal activity occurred almost exclusively during NREM sleep (about 98% of all episodes). A similar epileptiform activity was also detected by means of local field potential recordings during the 3rd postnatal week (15).

Given that the vast majority of SWDs in JAX mice occurred during slow-wave sleep, higher numbers of discharges might be expected during the hours mostly spent in SWS, but this is not what we observed. The fraction of time spent in SWS was highest during the central hours of the lights-on (resting) period and decreased rapidly to reach a minimum of 2–3 h after lights were turned off (27), corresponding to the period of highest behavioral activity (Figure 4C). Surprisingly, however, SWDs were relatively sparse during the early hours of the resting period but then increased and peaked sharply just before lights-off time (Figure 4B). Thus, periods of SWS taking place during late daytime and early nighttime hours, when animals are mostly active, were much more prone to epileptiform activity than SWS occurring during the periods of highest homeostatic pressure toward sleep (early and mid-daytime hours). For example, on Day 1, the number of SWDs per minute of time spent in SWS was 4 times higher between ZT11 and ZT12 (~0.4) than between ZT5 and ZT6 (~0.09). Importantly, this pattern of results was highly consistent across recording days 1 and 7, suggesting that the stable clinical picture shown in Figure 1C extends to the circadian rhythm of discharges. Finally, a similar circadian oscillation in the number of discharges can also be detected in a mouse model of cortical dysplasia-focal epilepsy, showing rare and short-lasting epileptic events similar to SWDs and peaking around lights-off time (42).

What makes SWS occurring during the first half of daytime more resilient to pathological discharges than later hours? Higher resistance to epileptiform discharges could correlate with low-frequency components of the EEG. It is well-known that in mice the spectral components in the delta frequency range (0.5–4 Hz), also known as slow-wave activity (SWA), are strongest in the first hours of rest and tend to decline later in the day, reaching a minimum around the light-to-dark shift and rising again during the dark hours (44–46). Experimental modulation of the amount and distribution of SWA during the 24 h might provide significant insights into this issue.

### Sleep alterations in JAX mice

Our analysis of the sleep–wake cycle in JAX mice revealed significant alterations compared to control animals. In particular, vigilance-state transitions were overall more frequent in JAX mice, and awakenings (transitions from NREM sleep to wakefulness) were especially increased. SWS was not only more fragmented (i.e., shorter duration of each bout), but also reduced in overall duration. Interestingly, we found that in animals with the most severe electrical alterations (higher number of SWDs) the sleep–wake cycle was also more fragmented. This correlation supports a causal relationship between SWDs and state changes in JAX mice. The hypothesis is further supported by the fact that state transitions were twice as likely to occur when preceded by SWD-containing epochs than by SWD-free epochs. As expected, the majority of such transitions consisted of awakenings.

The concept of state fragmentation has also been invoked with regard to the cortical dysplasia-focal epilepsy syndrome model (42). Specifically, the majority of SWDs in *Cntnap2* KO mice occurred during wakefulness, leading to a significant “fragmentation” of wakefulness compared to control animals. These observations suggest that SWDs impact the physiological alternation of vigilance states, regardless of the specific state in which discharges are concentrated. It should be noted, however, that the report in question (42) did not include a detailed description of the exact nature of such fragmentation or of the temporal relationship between individual SWD episodes and their effects on vigilance.

We have previously shown alterations of the sleep–wakefulness cycle also in the *Grin2a* KO model. Compared to WT controls, 30-day-old epileptic animals spent significantly less time in SWS and the number of state transitions was significantly higher, pointing to poor sleep quality, presumably related to the pathological EEG discharges.

Taken together, these results suggest a direct correlation between overall frequency and state-specific distribution of epileptiform activity, on the one hand, and the degree of disruption to the physiological alternation of vigilance states, on the other hand, which in turn is likely to have significant repercussions on behavioral and cognitive functions.

The consistent observation of vigilance state fragmentation across different mouse models, including the JAX mice and the Grin2a KO model, suggests that the presence of epileptiform activity is a key driver of sleep pathology. This disruption may be rooted in the shared neurobiological mechanisms underlying both SWD generation and sleep–wake regulation (47). It could be hypothesized that SWDs actively interfere with physiological sleep/wake alternation through direct perturbation of the thalamocortical circuitry. This circuit is critically involved in generating the slow oscillations characteristic of NREM sleep but is also the origin of SWDs (48). A dysregulated excitatory/inhibitory balance, favoring hyperexcitability, is central to this mechanism. Specifically, a reduction in effective GABAergic signaling or an increase in glutamatergic activity within the thalamic nuclei and cortex could simultaneously facilitate the onset of SWDs and disrupt the integrity of the sleep regulatory networks (49). The discharges, acting as powerful, intermittent bursts of abnormal synchronous activity, may acutely terminate or destabilize ongoing sleep states, mechanically accounting for the increased frequency of state transitions, particularly the observed increase in awakenings. This interpretation is supported by evidence highlighting that disturbances in the GABA/glutamate homeostasis are implicated not only in epileptogenesis but also in various sleep-related disorders, suggesting a pathophysiological convergence between seizure activity and poor sleep quality (50).

This framework could provide a direct causal link between the electrical pathology (SWDs) and the behavioral phenotype (sleep fragmentation and reduction in SWS duration), thereby justifying the correlation observed in JAX mice between SWD occurrence and degraded sleep quality.

The persistent presence of SWDs during sleep in humans is believed to cause brain damage, ultimately responsible for the cognitive and behavioral deficits observed in developmental syndromes such as LKS and CSWS, which is why these syndromes belong to the category of epileptic encephalopathies (EEs). While relatively rare, sleep-related EEs are typically drug-resistant and are probably under-detected due to the lack of clinical signs during the epochs of SWD-affected sleep.

Viable rodent models of EEs are of obvious translational importance, and the AJ JAX satisfies at least one requisite, i.e., the strong association between spontaneously occurring SWDs and SWS.

Several caveats must be pointed out, however. First, the human conditions are characterized by periods of continuous SWDs, typically occupying over 80% of the time spent in SWS. Compared to this pattern, however, our JAX mice showed a much sparser distribution of brief discharges. Whether this limited number of events is capable of determining translationally relevant cognitive and behavioral deficits is a question that requires direct experimental assessment.

The frequency of SWDs during sleep in affected children tends to decrease spontaneously and eventually disappear over the years (51, 52). Naturally, a candidate rodent model of the syndromes would be expected to follow a similar time-course.

Unlike our previous report of spontaneous SDWs detected by means of acute recordings of Grin2a^−/−^ mice during the 3rd postnatal week (15), the EEG recordings reported here were carried out in mice at 2–3 months of age, which in human terms corresponds to young adults. In this respect, the age range chosen for the present study is less than ideal. However, our focus was to carry out long-term epidural recordings in freely moving animals, which is unfeasible in animals of much younger age, due to the limited thickness of the skull and the overall brain size. Thus, with the current design, we are unable to establish the age of onset of discharges in JAX mice. We can, however, ask whether SWDs spontaneously disappear over time as they do in humans, and a study of similar design is currently in progress to address this question.

## Conclusion

**Figure 6:**
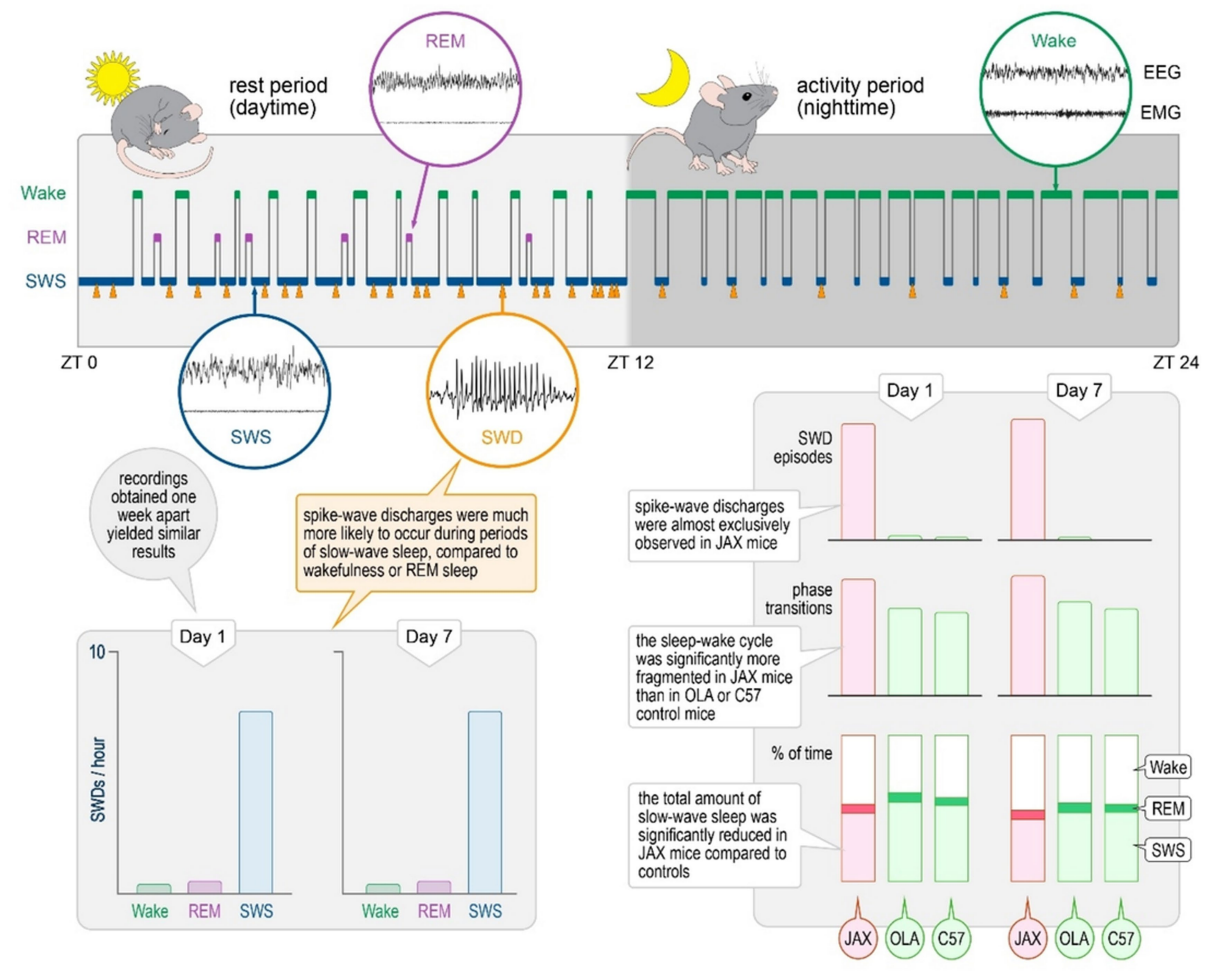
Summary of findings.

The present report confirms the consistent presence of spontaneously occurring SWDs during SWS in the AJ Jax inbred mouse strain and outlines a pattern of sleep disruption in affected animals. It also shows that, within SWS epochs, the frequency of SDWs varies during the circadian cycle. The relevance of these results is two-fold: (1) they provide a robust foundation for further molecular, electrophysiological, and simulation studies aimed at improving our understanding of the neural mechanisms of SWDs and their role as common denominators in a variety of conditions; (2) they pave the way for behavioral and cognitive experiments exploring possible associations between sleep-related discharges and observed deficits. The frequency of SWDs varies during circadian cycle (Figure 6).

## Data Availability

The raw data supporting the conclusions of this article will be made available by the authors, without undue reservation.

## Footnotes

1
https://www.jax.org/strain/000646

2
https://www.inotiv.com/model/a-jolahsd
