# Supplementary material for: Spontaneous spike-and-wave discharges during sleep in mice: circadian distribution and impact on sleep quality
Source: Front Neurol. 2026 Jan 16;16:1694773. doi: 10.3389/fneur.2025.1694773 (PMC12855067; doi:10.3389/fneur.2025.1694773)
Supplement: Supplementary file 1 [file Data_Sheet_1.PDF]

Supporting Information

Materials and methods

M1: Surgical procedures

Surgery was performed under sterile conditions and deep narcosis was induced and maintained by means of a dedicated gas anesthesia system (model no. 21200, Ugo Basile, Varese, Italy). Animals were placed in an induction chamber and deeply anesthetized with a mixture of isoflurane gas (4%, Merial, Ingelheim, Germany) and oxygen (~1.5 L/min, O_2). Once the absence of reflexes in hindlimbs and in *vibrissae* was verified, animals were removed from the induction chamber and anesthesia was maintained throughout the procedure with 1.5-2.5% of isoflurane in ~1.5 L/min of oxygen, administered through a mask for small rodents. Peripheral analgesic (carprofen, Rimadyl®, 1 mL/kg, 1:10) and a wide-spectrum antibiotic (enrofloxacin, Baytril®, 10 mL/kg, 1:50) were administered subcutaneously for prophylactic purposes. In addition, a drop of ophthalmic gel (Epigel, Ceva, Monza Brianza, Italy) was applied in each eye to protect and maintain the hydration of ocular bulbs. Animals were subjected to nuchal and cranial trichotomy and placed in a stereotaxic apparatus (Kopf Instruments, Tujunga CA, USA). After disinfection of the skin with Betadine® (Meda, Monza Brianza, Italy), two incisions were made in order to expose the left nuchal muscle and the skull.

Electromyographic (EMG) recordings were obtained by means of two Teflon-insulated, stranded, stainless-steel wires (Ø 0.1 mm, AS-631, Cooner Wire, Chatsworth, CA, USA). The tips of the electrodes were exposed, inserted into the left nuchal muscle, and fixed by means of suture thread (Ethilon 6-0, Ethicon, Sommerville NJ, USA). The wires' free leads were threaded subcutaneously to reach the cranial area.

Epidural, electroencephalographic (EEG) electrodes were represented by stainless-steel screws (M1, Ø 1.8 mm, length 2.23 mm) with an insulated copper wire soldered to the head of each screw. Four holes were first drilled through the skull in the right frontal, right parietal, left parietal, and occipital bones, at commonly adopted (1-3) stereotaxic coordinates (4). The screws were then threaded into each hole so that their tip would be firmly in contact with the *dura mater*.

Table S1.Stereotaxic coordinates for each electroencephalographic electrode. Values are indicated in mm and are referred to the distance from bregma. AP: antero-posterior; ML: medio-lateral.

Cranial Areas	Stereotaxic Coordinates	
	AP	*ML*
Right Frontal	+ 1.7	+ 1.5
Right Parietal	- 2.5	+ 2.0
Left Parietal	- 2.5	- 2.0
Occipital	- 5.8	0.0

All 6 electrodes (4 EEG + 2 EMG) were soldered to a 6-pin micro-connector and the entire implant was secured to the skull with dental cold-curing polymer resin. Finally, both skin incisions were sutured (Silk 4-0, Ethicon), and a wide-spectrum antibiotic spray (oxytetracycline hydrochloride, Terramicina®, Pfizer) was applied to the wounds. Analgesic (carprofen) and antibiotic (enrofloxacin) subcutaneous administrations were repeated for the following 2 and 3 days, respectively. Mice were allowed to recover from surgery for at least 14 days.

M2: In-vivo video-EEG/EMG recording setup

The Phenotyper (Noldus Information Technology®, Wageningen, The Netherlands) recording chambers were located in sound-attenuated rooms under the already mentioned

inverted light/dark cycle (ZT0: 6:00 pm) and controlled humidity and temperature (23 ±1 °C and 60 ±5% respectively).

The head implant was connected to a Grass Comet AS40 amplifier (Grass Technologies®, Natus Neurology Incorporated, Warwick RI) by means of an ultra-flexible cable with 6 leads (NMUF 6/30-4046SJ, Cooner Wire) carrying EEG and EMG signals. A mechanical arm (Multi-Axis Counter-Balanced Lever Arm SMCLA, Instech Laboratories Inc., Plymouth Meeting, PA, USA), fixed along the upper edge of the chamber, balanced the weight of the cable, thereby guaranteeing the animals' maximum freedom of movement.

M3: Sleep-wake analysis

Vigilance states were determined by visual scoring of the EEG (ipsilateral frontal-parietal derivation) and EMG traces in 10-s epochs using custom MATLAB® scripts (Mathworks, Natick MA, USA) which facilitated the generation of hypnograms. In addition, the software computed the power density estimation of EEG frequencies between 0 and 30 Hz, in 10-s epochs, based on Welch's overlapped-segment averaging estimator, with a 2-s moving Hanning window and 0.5-s overlap. The power spectra further aided the scoring process, especially with regards to the delta (0.5-4 Hz) and theta (6-9 Hz) components. Arousal states were defined as wakefulness (WAKE), slow-wave sleep (SWS) and rapid eye movement (REM) sleep based on changes in multiple parameters, including EEG and EMG appearance and EEG power density estimation. Briefly, WAKE was characterized by low-amplitude, mixed EEG frequency (delta and theta), and high muscle tone (MT). SWS was defined as high-voltage EEG, higher delta-band power compared to theta frequencies, and very low MT. REM was identified by low-amplitude EEG (wakefulness-like), with a peak in the theta frequency band and total absence of MT (5) Any movement artifacts or electrical noises in the recordings were tagged and the corresponding epochs excluded from subsequent analyses.

Results

R1: SWDs in the 3 groups

The table shows the number of mice that presented spike and wave discharges (SWDs), as well as the average number of SWDs (mean ± SEM) for each strain and during each day of recording.

Strain	Day of Recording			
	D1		D7	
	Affected Mice	n. discharges	Affected Mice	n. discharges
**JAX**	16/16	91.81 ± 9.99	16/16	94.94 ± 8.46
**OLA**	6/8	1.63 ± 0.53	5/8	1.63 ± 0.73
**C57**	4/16	0.69 ± 0.34	1/16	0.06 ± 0.06

Predictably, the mixed-model analysis of variance (MM-ANOVA) showed a highly significant effect of "strain" on the number of SWDs ($F_{2,37} = 81.94$, $p < 0.001$), but no effect of "day" ($F_{1,37} = 0.12$, $p = 0.736$) or interaction between factors ($F_{2,37} = 0.28$, $p = 0.755$) was observed. Tukey's HSD post-hoc tests revealed that the effect of "strain" was entirely attributable to the high number of SWDs in JAX mice (JAX vs. OLA $q = 13.40$, $p < 0.001$, JAX vs. C57, $q = 16.60$, $p < 0.001$), while no significant differences were found between OLA and C57 mice ($q = 0.18$, $p = 0.991$).

R2: Distribution of SWDs by vigilance state in JAX mice

A two-way (vigilance state*day) MM-ANOVA was performed on the number of SWD events occurring in each vigilance state. The test revealed a strong effect of "state" ($F_{2,30} = 97.20$, $p < 0.001$), but no effect of "day" ($F_{1,15} = 0.30$, $p = 0.595$) or interaction ($F_{2,37} = 0.28$, $p = 0.755$). Tukey's HSD post-hoc analysis showed that the "states" effect was due to highly significant differences in the mean number of SWDs in SWS compared to either WAKE (q =

16.8 , $p < 0.001$) or REM (q = 17.4 , $p < 0.001$), while no effect was observed between the two latter states (q = 0.57 , $p = 0.913$).

R3: Vulnerability of vigilance states to SWDs

Two separate MM-ANOVAs were carried out to evaluate the specific vulnerability of each state to the occurrence of SWDs, independent of the amount of time spent in them.

1) In terms of discharge numerosity, the effect of "vigilance state" was highly significant ($F_{2,30}$= 104.60, $p < 0.001$), where the events were more numerous during SWS than either REM (Tukey's HSD post-hoc test, q = 17.40, $p < 0.001$) or WAKE (q = 18.00, $p < 0.001$), which were affected to a similar degree (q = 0.56, $p = 0.916$). The counts did not differ between D1 and D7 (main effect of "day": $F_{1,15}$ = 0.85, $p = 0.370$) and no interactions were found ($F_{2,30}$ = 1.52, $p = 0.234$).

2) SWD duration also differed significantly between vigilance states (main effect of "state": $F_{2,30}$= 19.5, $p < 0.001$). In particular, Tukey's HSD post-hoc tests showed that SWDs lasted significantly longer in SWS compared to the other two states (REM: q = 8.66, $p < 0.001$; WAKE: q = 5.50, $p = 0.001$), and that discharges during WAKE lasted significantly longer than those in REM (q = 3.62, $p < 0.041$). Furthermore, the duration of SWDs significantly increased between D1 and D7 (main effect of "day": $F_{1,15}$ = 4.88, $p = 0.043$). This increase was independent of state, and no significant "state*day" interaction was detected ($F_{2,30}$ = 0.25, $p = 0.780$).

R4: Circadian distribution of SWDs

The easily discernible circadian pattern of SWD frequency was statistically confirmed as follows. The Rayleigh test for uniformity revealed that the distribution of SWD events during the 24h was not uniform (D1: Z_{Rayl} = 5.08, $p = 0.006$; D7: Z_{Rayl} = 7.24, $p = 0.001$), with

the previously mentioned peak around ZT12 (± 2:30 and ± 2:00 hours on D1 and D7, respectively). Further confirmation was obtained with the cosinor analysis applied to the frequency of SWDs over both D1 (mesor: 3.97, amplitude: 1.82 and acrophase: 11:59 h, $p < 0.001$) and D7 (mesor: 3.99, amplitude: 2.19 and acrophase: 11:54 h, $p < 0.001$).

R5: Total SWS duration

A significant between-group difference in total SWS ("strain" effect, $F_{2,37} = 12.00$, $p < 0.001$) was entirely due to the reduction of SWS in JAX mice, as shown by Tukey's HSD post-hoc tests (OLA: q = 5.79, $p < 0.001$; C57: q = 5.84, $p < 0.001$), whereas OLA and C57 mice did not significantly differ from each other (q = 1.02, p = 0.753). A moderate but significant reduction of total SWS was also observed at D7 compared to D1 ("day" effect, $F_{1,37} = 39.70$, $p < 0.001$). Such reduction was displayed by all 3 strains (Bonferroni's multiple comparisons test, C57 mice: t = 3.59, p = 0.003; OLA mice: t = 3.86, p = 0.001; JAX mice: t = 3.55, p = 0.003). No significant "strain*day" interaction was detected ($F_{2,37} = 0.72$, $p = 0.493$).

R6: Duration of SWS bouts

Significant between-group differences in the average duration of individual bouts of SWS were found (MM-ANOVA, effect of "strain", $F_{2,37} = 34.06$, $p < 0.001$), while no main effect of "days" ($F_{1,37} = 3.08$, $p = 0.087$) or strain*day interactions ($F_{2,37} = 0.50$, $p = 0.609$) were detected. The reduction in the duration of SWS episodes in JAX mice was entirely responsible for the effect of strain (Tukey's HSD post-hoc test, OLA mice: q = 7.14, $p < 0.001$; C57 mice: q = 11.40, $p < 0.001$). No differences between C57 and OLA mice were found (q= 2.21, $p = 0.276$).

R7: Frequency of SWS bouts

The average number of SWS bouts also changed significantly between strains (MM-ANOVA, effect of "strain": $F_{2,37} = 22.20$, $p < 0.001$), due to significantly higher number in JAX mice compared to either OLA (Tukey's HSD post-hoc test, q = 5.56, $p = 0.001$) or C57 mice (q = 9.21, $p < 0.001$), while the number of SWS episodes did not differ between control groups (q = 1.96, $p = 0.359$). No effect of "day" ($F_{1,37} = 0.81$, $p = 0.374$) or interaction ($F_{2,37} = 0.40$, $p = 0.672$) were found.

R8: Total REM duration

The total duration of REM did not change significantly between "strains" (MM-ANOVA, $F_{2,37} = 3.16$, $p = 0.054$) or "days" ($F_{1,37} = 0.03$, $p = 0.872$), and no significant "strain*day" interaction was found ($F_{2,37} = 2.24$, $p = 0.121$).

R9: Duration of REM bouts

A significant between-group difference in the average duration of REM bouts (MM-ANOVA, "strain" effect: $F_{2,37} = 8.27$, $p = 0.001$; "day" effect $F_{1,37} = 3.72$, $p = 0.062$; strain*day interactions, $F_{2,37} = 1.26$, $p = 0.296$) was entirely due to the reduction of REM bout duration in JAX compared to C57 mice, as shown by Tukey's HSD post-hoc tests (q = 11.00, $p < 0.001$), whereas JAX and OLA mice (q = 2.83, $p = 0.126$) as well as OLA and C57 mice (q = 1.85, $p = 0.400$) mice did not significantly differ from each other. No main effect of "day" ($F_{1,37} = 3.72$, $p = 0.062$) or strain*day interactions ($F_{2,37} = 1.26$, $p = 0.296$) were detected.

R10: Frequency of REM bouts

REM bouts were significantly more frequent in JAX mice (MM-ANOVA, effect of “strain”: $F_{2,37} = 8.47$, $p < 0.001$), than in C57 mice (Tukey’s HSD post-hoc test, q = 5.75, $p < 0.001$), while the number of REM episodes did not differ between JAX and OLA mice (q = 1.55, $p < 0.523$) or between control groups (q = 3.15, $p = 0.080$). No effect of “day” ($F_{1,37} = 0.21$, $p = 0.645$) or interaction ($F_{2,37} = 0.29$, $p = 0.753$) were found.

R11: Total WAKE duration

The analysis of the percentage of WAKE time revealed a significant effect of both “strain” (MM-ANOVA, $F_{2,37} = 10.01$, $p < 0.001$) and “day” ($F_{1,37} = 38.4$, $p < 0.001$), but no “strain*day” interaction was found ($F_{2,37} = 0.93$, p = 0.404). In particular, JAX mice spent more time awake compared to control animals (Tukey's HSD post-hoc test, C57 mice: q = 4.72, $p = 0.005$; OLA mice: q = 5.83, $p < 0.001$), whereas OLA and C57 mice did not significantly differ from each other (q = 1.97, $p = 0.353$). The significant effect of “day” was due to an increase in time spent in WAKE at D7 for all 3 strains (Bonferroni’s multiple comparisons test; C57: t = 3.67, $p = 0.002$; OLA: t = 3.90, $p = 0.001$; JAX: t = 3.19, $p = 0.009$).

R12: Duration of WAKE bouts

The total duration of WAKE did not change significantly between “strains” (MM-ANOVA, $F_{2,37} = 1.54$, $p = 0.228$) or “days” ($F_{1,37} = 2.55$, $p = 0.119$), and no “strain*day” interaction was found ($F_{2,37} = 2.65$, $p = 0.084$).

R13: Number of WAKE bouts

The average number of WAKE bouts changed significantly between strains (MM-ANOVA, $F_{2,37} = 10.01$, $p < 0.001$), due to a significantly higher frequency in JAX mice compared to both C57 (Tukey's HSD post-hoc test, q = 5.51, $p = 0.001$) and OLA mice (q = 5.15, $p = 0.002$), while the number of WAKE episodes did not differ between C57 and OLA mice (q = 0.65, $p = 0.892$). No effect of "day" ($F_{1,37} = 0.17$, $p = 0.678$) or interaction ($F_{2,37} = 0.86$, $p = 0.430$) were found.

R14: Total number of transitions between vigilance states

The analysis of the frequency of vigilance state transitions per hour (an inverse measure of sleep quality) revealed a significant main effect of "strain" (MM-ANOVA, $F_{2,37} = 22.43$, $p < 0.001$), due to significant higher number of vigilance state transitions in JAX mice than either C57 (Tukey's HSD post-hoc test: q = 6.41, $p < 0.001$) or OLA mice (q = 4.39, $p < 0.001$). The number of state transitions did not significantly change between recording days ($F_{1,37} = 1.84$, $p = 0.183$) and no "strain*day" interaction was detected ($F_{2,37} = 0.05$, $p = 0.948$).

R15: Number of pairwise transitions

While the above analysis does not differentiate between transition types, separate MM-ANOVAs and relative post-hoc tests were carried out to evaluate between- and within-group differences for each possible pair of vigilance states.

SWS to WAKE transitions: significant effect of "strain" ($F_{2,37} = 10.43$, $p < 0.001$). In particular, JAX mice show a higher number of awakenings than either OLA (Tukey's HSD post-hoc test: q = 5.12, $p = 0.002$) or C57 (q = 5.69, $p < 0.001$) mice, while no differences were

found between the latter (q = 0.47, *p* = 0.940). No effect of "day" ($F_{1,37}$ = 3.54, *p* = 0.068) or interaction ($F_{2,37}$ = 0.04, *p* = 0.959) were detected.

REM to WAKE transitions: main effect of "strain" ($F_{2,37}$ = 5.95, *p* = 0.006), due to relatively fewer of such transition in JAX mice compared to C57 mice (Tukey's HSD post-hoc test: q = 4.76, *p* = 0.005), while in this respect OLA mice did not differ from either JAX (q = 2.90, *p* = 0.114) or C57 mice (q = 0.98, *p* = 0.767). No effect of "day" ($F_{1,37}$ = 3.54, *p* = 0.068) or interaction ($F_{2,37}$ = 0.04, *p* = 0.959) were detected.

WAKE to SWS transitions: any significant effect of "strain" ($F_{2,37}$ = 1.15, *p* = 0.329), "day" ($F_{1,37}$ = 0.62, *p* = 0.435), or interaction "strain*day" ($F_{2,37}$ = 0.52, *p* = 0.598).

SWS to REM transitions: any significant effect of "strain" ($F_{2,37}$ = 0.36, *p* = 0.703), "day" ($F_{1,37}$ = 0.18, *p* = 0.685), or interaction "strain*day" ($F_{2,37}$ = 0.36, *p* = 0.703).

REM to SWS transitions: significant effect of "day" ($F_{1,37}$ = 14.80, $p < 0.001$), due to a higher percentage of such transition in D7 compared to D1 in C57 mice (Bonferroni's multiple comparisons test, t = 3.68, *p* = 0.002), no differences were found in both JAX (t = 0.924, *p* = 0.999) and OLA mice (t = 2.19, *p* = 0.104). No effect of "strain" ($F_{2,37}$ = 2.43, *p* = 0.500) or interaction ($F_{2,37}$ = 2.02, *p* = 0.147) were detected.

WAKE to REM transitions: statistics were not performed since these transitions are rare and were only occasionally observed in a subset of mice of all strains.

R16: Vigilance state transitions following SWDs

For each recording day, a Pearson's correlation coefficient showed a significant positive correlation between the number of observed SWDs and the frequency of state transitions (D1: ρ = 0.610, *p* = 0.012; D7: ρ = 0.659, *p* = 0.005).

The probability of observing a state transition immediately following an epoch containing one or more SWDs, compared with transitions that occurred after an SWD-free epoch was evaluated with paired t-tests (D1: t= 4.62, $p < 0.001$; D7: t= 6.28, $p < 0.001$).

References

1. Bouybayoune I, Mantovani S, Del Gallo F, Bertani I, Restelli E, Comerio L, et al. Transgenic fatal familial insomnia mice indicate prion infectivity-independent mechanisms of pathogenesis and phenotypic expression of disease. PLoS Pathog. 2015; 11(4):e1004796.

2. Jakubcakova V, Flachskamm C, Landgraf R, Kimura M. Sleep phenotyping in a mouse model of extreme trait anxiety. PLoS One. 2012; 7(7):e40625.

3. Mang GM, Franken P. Sleep and EEG Phenotyping in Mice. Curr Protoc Mouse Biol. 2012; 2(1):55–74.

4. Paxinos G, Franklin K. Mouse Brain in Stereotaxic Coordinates. Elsevier Science; 2012. 360 p.

5. Del Gallo F, Bianchi S, Bertani I, Messa M, Colombo L, Balducci C, et al. Sleep inhibition induced by amyloid-β oligomers is mediated by the cellular prion protein. J Sleep Res. 2021; 30(3):e13187.
