# Supplementary material for: Spontaneous spike-and-wave discharges during sleep in mice: circadian distribution and impact on sleep quality
Source: Front Neurol. 2026 Jan 16;16:1694773. doi: 10.3389/fneur.2025.1694773 (PMC12855067; doi:10.3389/fneur.2025.1694773)
Supplement: Supplementary file 2 [file Image_1.PDF]

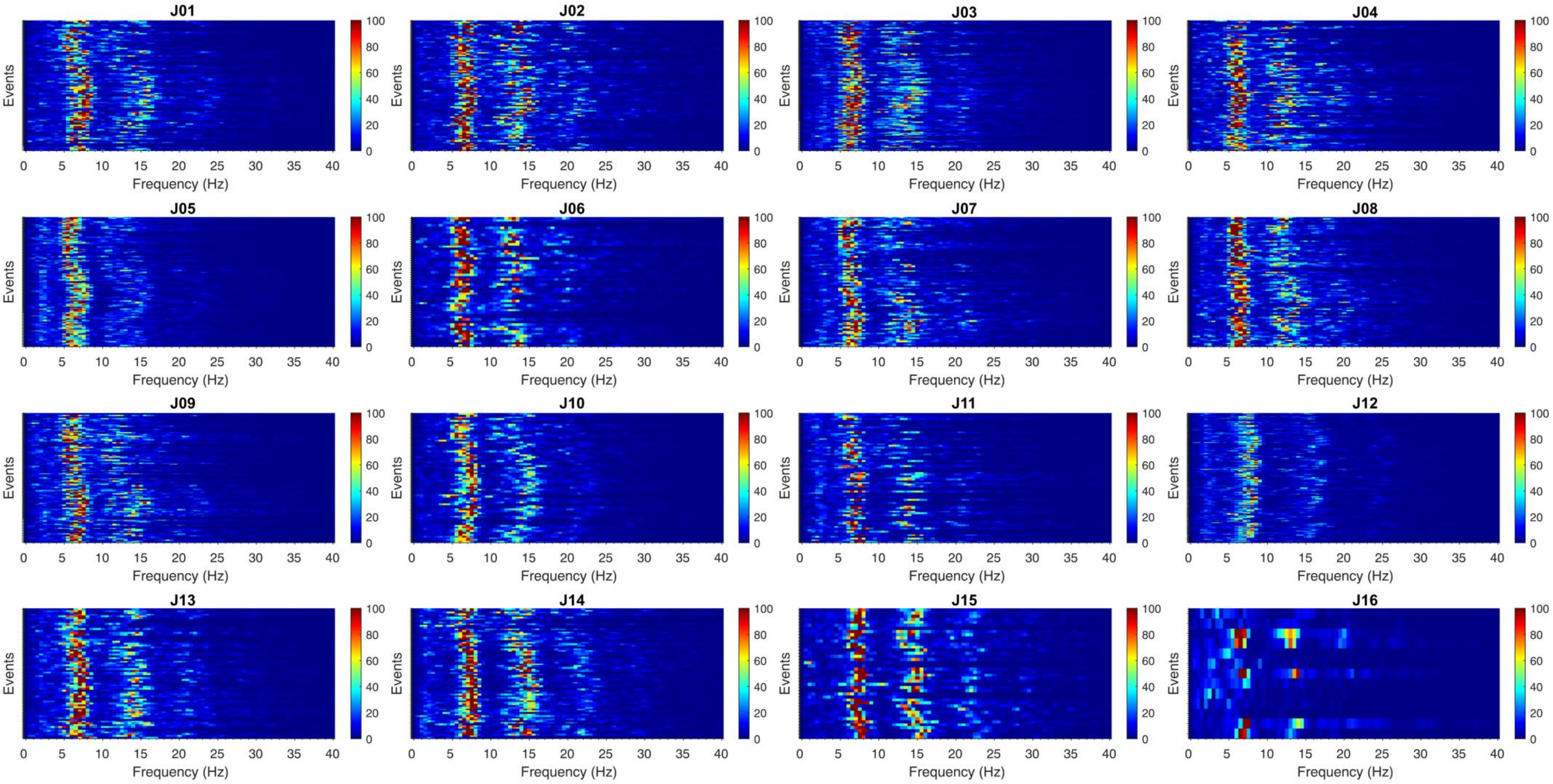

<u>Figure S1.</u> Day-1 SWD spectrograms. All detected discharges are shown (one row for each episode) for each recorded JAX mouse. Pseudocolors represent power values normalized for the minimum and maximum values observed in each animal.
